# Protocol for the analysis of proteasome activity, assembly state, and composition in fission yeast extracts using native gels

**DOI:** 10.1016/j.xpro.2025.104233

**Published:** 2025-12-04

**Authors:** Gabriel Ruiz-Romero, Rafael R. Daga, Silvia Salas-Pino

**Affiliations:** 1 Centro Andaluz de Biología del Desarrollo, Universidad Pablo de Olavide, Departamento de Biología Molecular e Ingeniería Bioquímica, 41013 Carretera de Utrera, Seville, Spain

**Keywords:** cell Biology, cell-based assays, cell separation/fractionation, protein Biochemistry

## Abstract

The proteasome is a macromolecular complex responsible for degrading short-lived or damaged proteins. Proteasome analysis presents challenges due to its high molecular weight and low stability. Here, we present a protocol to assess proteasome activity, assembly, and composition in *Schizosaccharomyces pombe*. We describe steps for lysate preparation and native electrophoresis to separate proteasome complexes. We then detail procedures for detecting proteasome activity using fluorescent substrates, fluorescently-tagged native proteins, and non-tagged proteasome components by immunodetection in a single gel.

For complete details on the use and execution of this protocol, please refer to Ruiz-Romero et al.^1^

## Highlights

• Protocol to analyze proteasome activity, assembly, and composition in a single native gel
• Steps for lysate preparation, native electrophoresis, and activity measurement
• Instructions to detect fluorescently tagged native proteasome subunits
• Steps for protein transfer and immunodetection of proteasome components

## Abstract (editor highlights)

Publisher’s note: Undertaking any experimental protocol requires adherence to local institutional guidelines for laboratory safety and ethics.

## Graphical abstract

**Figure:**
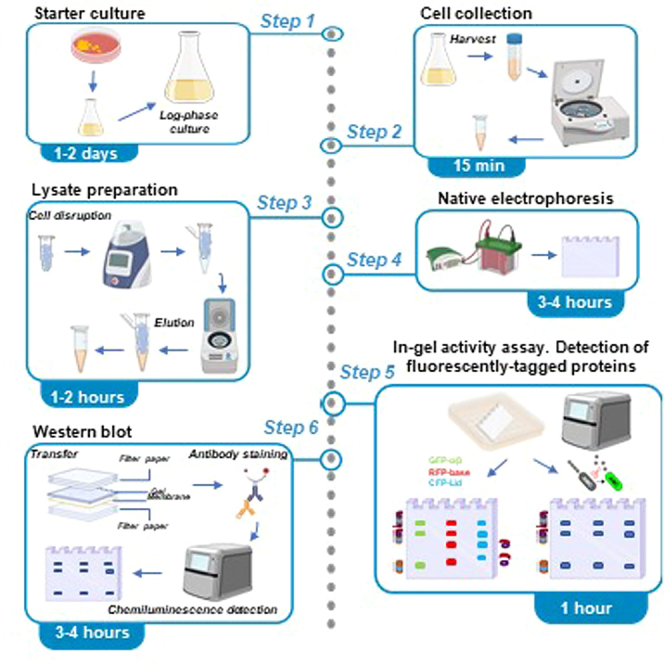

## Before you begin

This protocol is designed for use with *Schizosaccharomyces pombe*, but it was adapted from a protocol originally developed for *Saccharomyces cerevisiae*^2^ and can also be applied to that organism.

**Figure 1:**
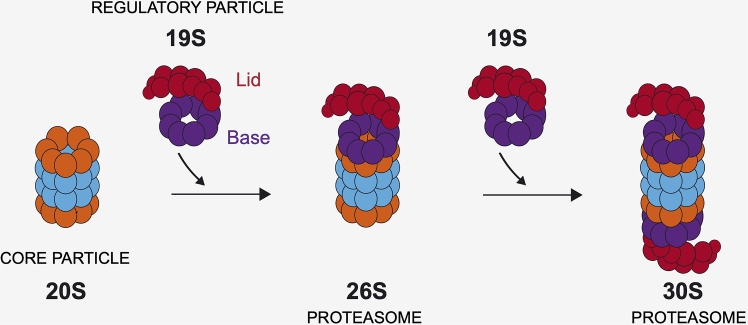
Main proteasome complexes The 20S proteasome CP can associate with one 19S Regulatory Particle (RP) to give rise to 26S proteasomes, or with two RP to give rise to 30S proteasomes. The 19S RP is composed of two subcomplexes: the base and the lid.

The proteasome is a ∼2.5 MDa macromolecular complex, composed of the 20S proteasome or Core Particle (CP), which associates with different regulators, the most abundant being the 19S Regulatory Particle (RP). CP can exist either as a free entity or in association with one or two RPs, giving rise to the 26S (RP-CP) and 30S (RP-CP-RP) proteasomes, respectively^3^^,^^4^^,^^5^ (Figure 1). In cells, proteasomes are found in a mixture of these different assembly states, with the free CP often reported as the most abundant form in various cell types.^6^ However, the relative abundance and activity of each proteasome species vary depending on cellular conditions. For instance, protein folding stress triggers the assembly of 26S/30S proteasomes, increasing their activity, while the activity of 20S proteasomes slightly decreases.^1^ Conversely, under oxidative stress, the dissociation of the 26S complex into free 20S and 19S subcomplexes is induced, favoring the conformation of the proteasome in the 20S state over the 26S state.^7^^,^^8^ Furthermore, in quiescent yeast cells experiencing carbon starvation, proteasomes exit the nucleus and concentrate in cytoplasmic foci named proteasome storage granules (PSGs), where the CP and RP are sequestered separately.^9^^,^^10^

Here, we describe a fast and reliable assay that allows the analysis of proteasome complexes in *S. pombe*, including their assembly state, activity and composition without the need for previous proteasome purification. In this protocol, *S. pombe* lysates are subjected to native gel electrophoresis, followed by incubation with the fluorogenic substrate SucLLVY-AMC, which emits fluorescence upon cleavage by the chymotrypsin-like activity of the proteasome.^11^ This protocol also enables the identification and characterization of novel proteasome species, such as the intermediate proteasome (i-P) recently identified in *S. pombe*, using fluorescently tagged proteasome subunits and associated proteins, which can be visualized directly in native gels.^1^ Alternatively, untagged proteasome subunits can be detected by immunoblotting with specific antibodies upon transfer of proteins from the gel to an appropriate membrane.

### Innovation

This protocol describes an optimized method to determine proteasome activity using native gel electrophoresis and fluorescent proteasome substrates specifically adapted for *Schizosaccharomyces pombe*. While similar approaches have been applied to *Saccharomyces cerevisiae* and human cells, this is the first protocol optimized for *S. pombe*, thereby expanding the applicability of native gel-based assays to this model organism. Unlike conventional protocols that rely on previous proteasome purification, this method uses total cell lysates, minimizing sample manipulation and reducing the time and amount of starting material. The workflow is rapid, reproducible and suitable for parallel comparison across *S. pombe, S. cerevisiae* and human cells. A key advancement of this protocol is the integration of proteasome activity assays with immunodetection and visualization of fluorescently-tagged endogenous proteins. This combined approach enables the analysis of proteasome activity, composition and assembly state under native conditions in a single experiment.

## Key resources table

**Table:** 

REAGENT or RESOURCE	SOURCE	IDENTIFIER
**Antibodies**		

Proteasome 20S α1, 2, 3, 5, 6 & 7 subunits monoclonal (working dilution: 1:2,000)	Enzo Life Sciences	MCP231
Goat antibody anti-mouse IgG (working dilution: 1:2,000)	Sigma-Aldrich	A3562; RRID: AB_258091

**Chemicals, peptides, and recombinant proteins**		

Acid-washed glass beads	Sigma-Aldrich	G9268
Tween 20	Sigma-Aldrich	P7949
Disuccinimidyl suberate, cross-linking reagent (DSS)	Abcam	141274
30% acrylamide/bis solution, 37.5:1	Bio-Rad	1610158
Adenosine 5′-triphosphate disodium salt hydrate (ATP)	Biosynth	NA00135
Dithiothreitol (DTT)	Thermo Fisher Scientific	R0862
Suc-Leu-Leu-Val-Tyr-AMC	Cayman Chemical	10008119
Sodium dodecyl sulfate (SDS)	Sigma-Aldrich	L6026
Bromophenol Blue sodium salt	Sigma-Aldrich	B5525
Ammonium persulfate (APS)	Sigma-Aldrich	A3678
N,N,N′,N′-tetramethylethylenediamine (TEMED)	Sigma-Aldrich	T22500
Non-fat dry milk	Nestlé	N/A
Sucrose	Sigma-Aldrich	S9378
Dimethyl sulfoxide (DMSO)	Sigma-aldrich	D8418
Yeast extract	Condalab	1702.00
Leucine	Sigma-aldrich	L8000
Lysine	Sigma-aldrich	L5501
Uridine	PanReac AppliChem	A0666,0025
Adenine	Thermo Scientific	A17622.22
Histidine	PanReac AppliChem	A3733,0100
Glucose	PanReac AppliChem	131341.0416
NaCl	PanReac AppliChem	131659.1211
KCl	PanReac AppliChem	131494.1214
Na_2_HPO_4_	PanReac AppliChem	131679.1211
KH_2_PO_4_	PanReac AppliChem	131509.1211
Glycerol	Sigma-aldrich	G5516
Magnesium chloride	Sigma-aldrich	M8266
Tris base	Merck	10708976001
Boric acid	Sigma-aldrich	B0394
EDTA	Sigma-aldrich	E9884

**Critical commercial assays**		

SuperSignal West Femto maximum sensitivity substrate	Thermo Fisher Scientific	34095
Mini-PROTEAN Tetra handcast systems	Bio-Rad	1658000
Trans-Blot Turbo transfer system	Bio-Rad	1704150
Pierce BCA protein assay kit	Thermo Fisher Scientific	23225

**Experimental models: Organisms/strains**		

*S. pombe wild type* strain 972 h-	Laboratory stock	RD1937

**Software and algorithms**		

Image Lab Software	Bio-Rad	https://www.bio-rad.com/es-es/product/image-lab-software?ID=KRE6P5E8Z

**Other**		

UV-1280 spectrophotometer	Shimadzu	https://www.shimadzu.com/an/products/molecular-spectroscopy/uv-vis/uv-vis-nir-spectroscopy/uv-1280/index.html
Amersham Protran Premium western blotting membranes, nitrocellulose	Sigma-Aldrich	GE10600003
ChemiDoc	Bio-Rad	https://www.bio-rad.com/es-es/product/chemidoc-imaging-system?ID=OI91XQ15
FastPrep-24 5G beating grinder and lysis system	MP Biomedicals	https://www.mpbio.com/us/fastprep-24-5g-instrument

## Materials and equipment

**Table:** YES medium (liquid)

Reagent	Final concentration	Amount
Yeast extract	5 g/L	5 g
Leucine	0.3 g/L	0.3 g
Uridine	0.3 g/L	0.3 g
Lysine	0.3 g/L	0.3 g
Adenine	0.3 g/L	0.3 g
Histidine	0.3 g/L	0.3 g
ddH_2_O	N/A	Up to 600 mL

Sterilize by autoclaving

**Table:** 

Glucose (75 g/L)	30 g/L	400 mL
**Total**	**N/A**	**1,000 mL**

Autoclave yeast extract plus supplements and glucose solution separately and then mix both solutions. Store at room temperature for up to 6 months.

**Table:** PBS buffer

Reagent	Final concentration	Amount
NaCl	8 g/L	8 g
KCl	0.2 g/L	0.2 g
Na_2_HPO_4_	1.44 g/L	1.44 g
KH_2_PO_4_	0.24 g/L	0.24 g
ddH_2_O	N/A	Up to 1,000 mL

Adjust pH to 7.5 by adding NaOH.

**Table:** 

**Total**	**N/A**	**1,000 mL**

Sterilize by autoclaving. Store at room temperature for up to 12 months.

**Table:** Protein Extraction Buffer

Reagent	Final concentration	Amount
Tris-HCl, pH 7.5 1 M	50 mM	10 mL
MgCl_2_ 1 M	5 mM	1 mL
Glycerol 100% (v/v)	15% (v/v)	30 mL
ddH_2_O	N/A	156.8 mL

Storage at room temperature for up to 3 months.

**Table:** 

Reagent	Final concentration	Amount
ATP 200 mM	2 mM	2 mL
DTT 1 M	1 mM	0.2 mL
**Total**	**N/A**	**200 mL**

The stock of ATP 200 mM can be stored in small aliquots at −80°C for up to 6 months. The stock of DTT 1 M can be stored in small aliquots at −20°C up to 6 months. Thaw the ATP and DTT stocks in ice. Add the volume of ATP stock and DTT stock needed for the volume of Protein extraction buffer required for each experiment right before use.

**Table:** DSS stock

Reagent	Final concentration	Amount
Disuccinimidyl suberate, cross-linking reagent (DSS)	25 mM	0.092 g
DMSO	N/A	1 mL
**Total**	**N/A**	**1 mL**

Prepare before use. Do not store.

**Table:** TBE buffer 10×

Reagent	Final concentration	Amount
Tris Base	890 mM	108 g
Boric acid	890 mM	55 g
EDTA	2.5 mM	7.4 g
ddH_2_O	N/A	Up to 1,000 mL
**Total**	**N/A**	**1,000 mL**

Adjust pH to 8.3 and sterilize by autoclaving.

Store at room temperature for up to 1 year.

**Table:** 4× Loading buffer

Reagent	Final concentration	Amount
Tris-HCl 1 M, pH 6.8	200 mM	3 mL
Glycerol	60% (v/v)	9 mL
Bromophenol blue	6 mM	60.3 mg
ddH_2_O	N/A	Up to 15 mL
**Total**	**N/A**	**15 mL**

Store at −20°C.

**Table:** 4% Polyacrylamide running gel

Reagent	Final concentration	Amount
TBE 10×	1×	1.5 mL
30% Acrylamide/Bis Solution, 37.5:1	4%	2 mL
Sucrose 10% (w/v)	2.5% (w/v)	3.75 mL
MgCl_2_ 1M	5 mM	0.075 mL
DTT 1M	5 mM	0.075 mL
ATP 200 mM	1 mM	0.075 mL
ddH_2_O	N/A	7.465 mL
APS 10%	0.03%	0.05 mL
TEMED	0.066%	0.01 mL
**Total**	**N/A**	**15 mL**

Prepare before use. Do not store.

**Table:** Stacking gel

Reagent	Final concentration	Amount
Tris-HCl 1 M pH 6.8	50 mM	0.5 mL
30% Acrylamide/Bis Solution, 37.5:1	2.49%	0.83 mL
ddH_2_O	N/A	8.61 mL
APS 10%	0.05%	0.05 mL
TEMED	0.1%	0.01 mL
**Total**	**N/A**	**10 mL**

Prepare before use. Do not store.

**Table:** Electrophoresis buffer

Reagent	Final concentration	Amount
Tris base	90 mM	10.90 g
Boric acid	80 mM	4.94 g
EDTA	0.1 mM	0.04 g
MgCl_2_	5 mM	0.48 g
ddH_2_O	N/A	Up to 1,000 mL

Storage at room temperature for up to 6 months.

**Table:** 

Reagent	Final concentration	Amount
ATP	1 mM	0.55114 g
DTT 1 M	1 mM	1 mL
**Total**	**N/A**	**1, 000 mL**

Add ATP and DTT right before use.

**Table:** Proteasome substrate stock solution

Reagent	Final concentration	Amount
Suc-Leu-Leu-Val-Tyr-AMC	13 mM	10 mg
DMSO	N/A	1 mL

Store at −20°C protected from light. Store for up to two weeks.

## Step-by-step method details

### Cell culture

**Timing: 1–2 days**

Cells are cultured under optimal conditions in liquid medium.

1. Inoculate a small amount of cells into 5–10 mL of liquid YES medium (or minimal medium, as required) in a 25–50 mL Erlenmeyer flask.
2. Incubate the culture at 25°C–30°C with shaking at 180 rpm for approximately 12–16 h.
3. Dilute the culture into 50–100 mL of liquid YES medium.
4. Continue incubation until the culture reaches mid-log phase (OD_595_: 0.4–0.6), measured using a Shimadzu UV-1280 spectrophotometer or a similar instrument.

***Note:*** Proteasome expression and assembly can vary considerably depending on the culture OD. For consistency, cells should always be collected at a similar OD around mid-log phase.

### Cell collection

**Timing: 15 min**

Cells are harvested, washed with PBS and pelleted in a 1.5 mL screw-cap tube for subsequent cell disruption.

5. Centrifuge the cells at 25°C, 1,740 × *g* for 5 minutes. Discard the supernatant.

***Note:*** Cultures are centrifuged only after reaching mid-log phase. If any treatment is required, such as the inactivation of thermosensitive mutants or the addition of chemicals, the time of incubation should be taken into account to obtain the final optical density.

6. Resuspend the pellet in 1 mL of ice-cold PBS buffer and transfer the cells to a 1.5 mL screw-cap tube.
7. Centrifuge at 4°C for 4 minutes at 1,740 × *g*.
8. Discard the supernatant and snap-freeze the cell pellet in liquid nitrogen.

***Note:*** Samples can be stored at −80°C at this point. Alternatively, proceed with the next steps.

### Lysate preparation

**Timing: 1–2 h**

Cell lysates are obtained by mechanical disruption with glass beads. All steps should be performed on ice.

9. Add 240 μL of Protein Extraction Buffer to the frozen cell pellet and let it thaw on ice. Resuspend the pellet thoroughly using a pipette.

***Note:*** If DSS is not to be added (step 10), use 300 μL of Protein Extraction Buffer.

10. Add 60 μL of the lysine-reactive crosslinker disuccinimidyl suberate (DSS) to achieve a final concentration of 5 mM. Mix immediately.

***Note:*** DSS addition stabilizes 26S/30S particles by inhibiting partial dissociation and improves band resolution in native gels. Although optional, this step is especially beneficial when glass beads are used for cell disruption.

11. Add acid-washed glass beads until the entire volume is covered. Mix well by inverting the tube.

***Note:*** Do not fill the screw-tap tube entirely with glass beads, as this can hinder effective cell disruption.

12. Cell lysis.
  a. Lyse the cells using a FastPrep (MP Biomedicals) with four 20-second pulses, speed 6, keeping the samples on ice for 1 minute between pulses.
  b. Verify successful cell disruption by examining a sample under the microscope with 2–3 μL of 10% SDS. A simple quantitative estimate can be obtained by counting the number of intact versus lysed cells in the field of view before and after lysis (70%–80% of cell disruption is recommended).

***Note:*** Intact cells are resistant to low concentrations of SDS; however, lysed or partially lysed cells are not, and they lose their shape and release their contents upon SDS addition.

***Note:*** Cells can also be disrupted using a mortar and pestle and liquid nitrogen, which often yields slightly cleaner results than disruption with the FastPrep. However, this method is time-consuming, especially when handling large number of samples.

13. Elute the sample.
  a. Flame the tip of a needle and use it to poke a hole at the bottom of the tube containing the cell lysate.
  b. Place the tube over a clean Eppendorf tube and centrifuge at 3,300 × *g* for 30 seconds to collect the eluate.
14. Clear the eluate by centrifuging at 15,500 × *g* for 10 minutes at 4°C. Transfer the clarified supernatant to a new Eppendorf tube. Repeat this step twice.

***Note:*** Protein samples can be stored at −80°C if needed. However, repeated freeze-thaw cycles may disrupt the stability of proteasome complexes. For consistency, we recommend avoiding freezing the samples.

15. Determine protein concentration using the Pierce BCA Protein Assay Kit (23225, Thermo Fisher Scientific).

### Native electrophoresis

**Timing: 3–4 h**

Protein samples are separated on a native gel.

16. Adjust all protein samples to the same total protein concentration by adding protein extraction buffer when needed. Add ¼ of the total sample volume of 4× Loading buffer, then resuspend gently.

***Note:*** Protein samples can be stored at −80°C at this point to pause the protocol. Saving aliquots is recommended to avoid multiple freeze/thaw cycles.

17. Prepare the running gel.
  a. Clean 1.5 mm spacer electrophoresis glass plates with water (do not use ethanol, as residual ethanol may cause the gel to stick to the glass, leading to breakage during separation). Dry the plates thoroughly, either by air drying or using filter paper.
  b. Assemble the gel casting cassette.
  c. Prepare the running gel by mixing its components and pour it between the glass plates.
  d. Add a 2–3 mm layer of isopropanol on the top and allow the gel to solidify for at least 30 minutes.
  e. Remove isopropanol by pouring it off, then wash three times with ddH_2_O. Carefully remove any remaining water with a piece of filter paper, without touching the gel surface

***Note:*** Using 1.5 mm spacer glass plates is recommended, as thinner plates (1.0 mm spacers) increase the risk of gel breakage.

***Note:*** The running gel contains 4% acrylamide and 2.5% sucrose, which increases gel density and improves the resolution of large protein complexes.

***Note:*** The indicated volumes of running and stacking gel solutions are sufficient to prepare two gels.

18. Prepare the stacking gel (2.49% acrylamide).
  a. Mix the components of the staking gel and pour it on top of the running gel.
  b. Insert a clean 10–15-well plastic comb and allow it to solidify for at least 30 minutes.
19. Assemble the gel into the electrophoresis chamber and fill it with electrophoresis buffer.
20. Carefully remove the comb and load the protein samples into the wells.
21. Run the electrophoresis at 150 V for 80 minutes in a cold room at 4°C. Alternatively surround the electrophoresis chamber with ice to maintain it cool. This prevents the gel from melting during the running.

***Note:*** 80 minutes is generally sufficient to separate proteasome complexes. For additional separation, longer run times may be used, but this may reduce band resolution.

### In-gel activity assay: Detection of fluorescently tagged proteins

**Timing: 1 h**

After electrophoresis, the gel is incubated in a solution containing a proteasome substrate, which releases fluorescence upon cleavage. The fluorescence produced allows detection and identification of active proteasome complexes.

22. Pour 10 mL of electrophoresis buffer (the same buffer used during electrophoresis can be reused) into a small container, slightly larger than the gel.
23. Carefully separate the electrophoresis glass plates and place the gel into the container with the electrophoresis buffer.
24. Add 100 μL of proteasome substrate stock solution to achieve a final concentration of 0.130 mM and mix by gentle agitation. Cover the container with aluminum foil to keep its interior dark. Incubate the gel at 36°C for 15 minutes with gentle shaking (no more than 100 rpm).

***Note:*** Thaw a 100 μL aliquot of the proteasome substrate stock solution in the dark. Avoid exposure to light.

***Note:*** Incubation is typically performed at 30°C, although 36°C can also be used for short-term assays to obtain rapid and reproducible results.

***Note:*** SucLLVY-AMC is the most commonly used substrate for detecting proteasome activity. It is specifically cleaved by the chymotrypsin-like activity of the proteasome. Other fluorogenic substrates can be used to assess the additional trypsin-like or caspase-like proteolytic activities of the proteasome.

**CRITICAL:** Image the fluorescence immediately after incubation with the substrate. Avoid storing the gel for analysis on subsequent days, as the fluorescence of the bands diminishes over time due to diffusion of the fluorescent AMC group from the sites of proteasomal cleavage. This leads to reduced band resolution and intensity.

**Figure 2:**
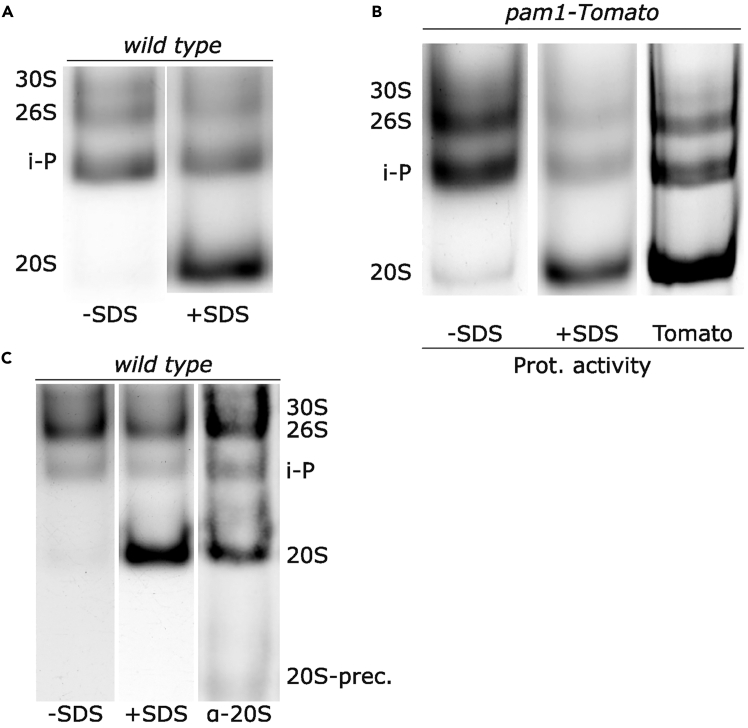
In-gel proteasome analysis (A) In-gel proteasome activity assay of extracts from wild-type cells grown at 25°C. Left lane: in-gel assay without SDS. Right lane: same gel imaged after incubation with 0.04% SDS, which opens and activates the 20S proteasome. (B) Left and middle lanes: in-gel proteasome activity assay of cells expressing Pam1-Tomato (before and after SDS addition). Right lane: Pam1-tomato signal detection in the same gel. (C) Left and middle lanes: in-gel proteasome activity assay of wild-type cells (before and after SDS addition). Right lane: the same gel was immunoblotted with anti-α1-7 antibody.

25. Assess proteasome activity by imaging the fluorescence signal using a ChemiDoc MP Imaging System (Bio-Rad) with the Oriole Method in Image Lab software.
26. Place the gel back into the container with electrophoresis buffer and substrate, then add 20 μL of SDS 20% to reach a final concentration of 0.04%, which facilitates the opening and activation of 20S proteasomes^12^ (Figure 2A). Incubate at 36°C for another 15 minutes with gentle shaking and re-analyze activity using the same imaging program.
27. For visualizing fluorescently-tagged proteins, use alternate settings on the ChemiDoc MP Imaging System (Bio-Rad): select the Pro-Q Emerald 488 method for GFP visualization, or Pro-Q Diamond for Td-Tomato/RFP visualization (Figure 2B).

***Note:*** This step is independent of the activity analysis and can be performed before or after assessing activity, as well as before or after adding SDS.

**CRITICAL:** Fluorescence visualization requires the use of strains expressing fluorescently-tagged proteasome subunits or regulators.

### Western blot analysis

**Timing: 3–4 h**

Proteins are transferred from the gel onto a membrane and probed with an appropriate antibody. Using an anti-20S antibody, enables the detection of all proteasome species, including proteasome subparticles lacking activity (Figure 2C). Additionally, antibodies against other subunits can be used as an alternative or in combination with fluorescently tagged proteins.

28. Transfer the proteins from the gel to a nitrocellulose membrane using the Trans-Blot Turbo Transfer System (Bio-Rad). Use the standard SD protocol, which involves transferring at a constant 25 V and no more than 1 A for 30 minutes.

***Note:*** Users should be aware that cross-linking can potentially influence transfer. In our experimental conditions, cross-linking with DSS does not seem to significantly affect the transfer efficiency of proteasome complexes.

29. Block the membrane by incubation in PBS containing 0.1% Tween 20 (PBST) and 10% (w/v) non-fat dry milk, for 10–15 minutes.
30. Incubate the membrane with anti α1, 2, 3, 4, 5 & 7 subunits antibody (anti-20S; MCP231; Enzo Life Sciences) at a 1:2000 dilution in PBST with 5% (w/v) non-fat dry milk. Incubate for 1–2 h at room temperature or overnight at 4°C.

***Note:*** The suitability of each antibody should be empirically tested under the specific conditions of this protocol.

31. Wash the membrane for 5 minutes with PBST with gentle agitation. Repeat this step twice.
32. Incubate the membrane with a secondary anti-mouse IgG antibody (A3562; Sigma-Aldrich) at a 1:2000 dilution in PBST plus 5% (w/v) non-fat dry milk. Incubate at room temperature for 1 hour.
33. Wash the membrane for 5 minutes with PBST with gentle agitation. Repeat this step twice.
34. Finally, proceed with protein detection using the Supersignal West Femto substrate (34095, Thermo Fisher) and the ChemiDoc MP Imaging System (Bio-Rad) with the Chemiluminescence Method in Image Lab software.

## Expected outcomes

The in-gel proteasome activity assay allows the detection of different proteasome species with catalytic activity. Loading between 50 and 70 μg of proteins is generally sufficient to detect these bands. Incubation of the gel with 0.04% SDS results in an increase in activity of 20S proteasomes (Figure 2A), which facilitates their identification in the gel.

While immunoprecipitation of proteasomes using antibodies against 19S subunits only enables the detection of proteasome species containing the 19S Regulatory Particle, analyzing proteasomes directly from whole cell extracts allows the identification of all proteasome species present in the sample (Figure 2C). This approach allowed the detection of the intermediate proteasome species in *S. pombe*^1^ (Figure 2A), which had previously been overlooked due to the predominant use of immunoprecipitation targeting 19S subunits for proteasome analysis.

The composition of each proteasome band detected in the gel can be studied by analyzing the colocalization of the fluorescence from tagged proteins and the bands obtained in the activity assay within the same gel (Figure 2B). Alternatively, the use of specific antibodies against proteasome components by immunoblotting after protein transfer to a suitable membrane allows assessment of band composition in untagged strains (Figure 2C). Furthermore, both approaches facilitate the identification of proteasome bands lacking catalytic activity—such as the 19S Regulatory Particle and its precursors, as well as 20S precursors—which would otherwise be missed by activity-based assays. This method has proven suitable for studying global changes affecting proteasome composition and assembly under different conditions. For instance, folding stress induced either by heat shock or by the addition of amino acid analogs has been shown to promote the assembly of 26S/30S proteasomes from preexisting precursors,^1^ such as free 20S cores. Likewise, inactivation of *ump1-ts*, a mutant defective in 20S proteasome assembly, leads to a decrease in 20S proteasomes and the accumulation of low molecular weight precursors which are detected with an anti 20S antibody in native gels.^1^

## Limitations

One limitation of this assay is the difficulty in finding a suitable molecular weight marker and loading control due to the large size of proteasome complexes. As an alternative for a loading control, a denaturing western blot can be performed using the same volume of samples used in the native gel to detect a housekeeping protein.

For the analysis of proteasome precursor complexes, which might be of small size and lack catalytic activity, either the use of tagged strains or a western blot following a short-run native gel electrophoresis should be employed. However, this short electrophoresis can compromise the separation and resolution of bands corresponding to higher proteasome species. As a result, it may be necessary to perform two separate electrophoresis runs with corresponding western blots, depending on the proteasome species or complexes to analyze.

## Troubleshooting

### Problem 1

Proteasomes disassembly. Absence of 26S/30S proteasome bands. Related to protein extraction and native electrophoresis running.

**Figure 3:**
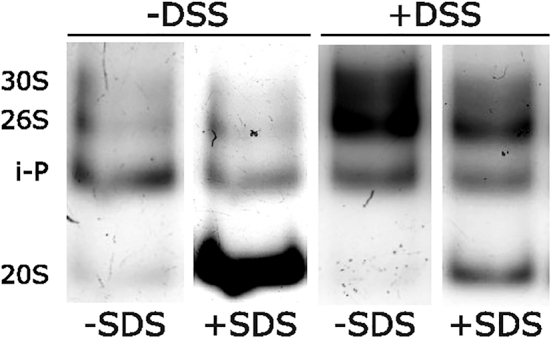
Effects of DSS Left panels: in-gel activity assay of extracts from wild-type cells prepared using extraction buffer without DSS. Right panels: same assay using extracts prepared with the same buffer supplemented with DSS. In both cases, the gel was imaged before and after incubation with 0.04% SDS.

### Potential solution

• Proteasomes from *S. pombe* and other species may be less stable than those from *S. cerevisiae* or humans. We recommend the use of chemical cross-linkers, such as formaldehyde or DSS (see step 10), to prevent partial dissociation and improve band resolution, providing more reliable results (Figure 3).
• Excessive cell disruption can result in protein degradation. Avoid disrupting more than 70%–80% of the cells (see step 12).
• Proteasomes disassemble into 20S and 19S subparticles in the absence of ATP. We recommend to aliquot ATP stocks or use fresh ATP for each experiment.

### Problem 2

The acrylamide gel loses its integrity after running. Related to step 22. Native electrophoresis.

### Potential solution

The acrylamide gel may overheat and soften or lose integrity during running. To avoid this always run the gel in a cold room at 4°C or surround the gel chamber with ice during running.

### Problem 3

Gel breakage. Related to step 24.

### Potential solution

The low acrylamide percentage used makes the gel highly prone to breaking. We recommend using a wedge spatula or a similar instrument to carefully lift and transfer the gel. Avoid lifting the gel from only one side, as its own weight can cause it to tear. Instead, support the entire gel from underneath when handling.

### Problem 4

Weak or absent signal after immunoblotting. Related to step 31. Western blot analysis.

### Potential solution

Not all antibodies are compatible with immunoblotting after native PAGE. Antibodies recognize specific epitopes which might be native or denatured. In case of weak or absent signal after immunoblotting consider testing alternative antibodies.

## Resource availability

### Lead contact

Further information and requests for resources and reagents should be directed to and will be fulfilled by the lead contact, Silvia Salas-Pino (ssalpin@upo.es).

### Technical contact

Gabriel Ruiz Romero (gruirom@upo.es), Silvia Salas Pino (ssalpin@upo.es).

### Materials availability

This study did not generate new unique reagents.

### Data and code availability

The published article Ruiz-Romero et al.^1^ includes all datasets analyzed during this study.

## Acknowledgments

We would like to thank Victor M. Carranco (Genetics area) and Laura Tomás (CABD proteomic facility) for excellent technical help. We thank Mark Hochstrasser for useful discussions. This work has been funded by MICIU/AEI/10.13039/501100011033/, FEDER UE, grant numbers PID2021-128408OB-I00 and PID2024-160582OB-I00 to R.R.D. G.R.-R. is funded by fellowship FPU, ref.: FPU17/02201, Ministerio de Ciencia, Innovación y Universidades, Spain.

## Author contributions

G.R.-R. conducted and analyzed experiments and wrote the manuscript. R.R.D. supervised the study and acquired funding. S.S.-P. conducted and analyzed experiments, reviewed and edited the manuscript, and supervised the study.

## Declaration of interests

The authors declare no competing interests.
